# Left Inguinal Colotomy for Carcinoma of Rectum

**Published:** 1890-01

**Authors:** J. McF. Gaston


### Reports of Cases.

### LEFT INGUINAL COLOTOMY FOR CARCINOMA OF RECTUM, PERFORMED BY J. McF. GASTON, M. D.

Reported for the JOURNAL.

A white man, thirty-eight years old, came under the care of Dr. J. McF. Gaston, at the Providence Infirmary, during the month of November, 1889, for a growth in the rectum. After the use internally of Fowler's Arsenical solution, with Succus Alterans for several weeks, and the local application within the rectum of a mixture of iodoform with balsam of Peru daily without improvement of the induration, colotomy was considered proper to remove the increasing impediment to fecal evacuations.

Accordingly, at the regular surgical clinic, on December 4th, Dr. Gaston, with the assistance of Dr. W. P. Nicolson, proceeded to make an incision, running in the line from the anterior superior spinous process of the ilium to the spine of the pubic bone, into the peritoneal cavity. The surface of the sigmoid flexure of the colon presented in the wound, and its serous and muscular coats were attached by continuous iron dyed silk suture to borders of the incision, bringing the serous parietal membrane in contact with that of the intestine, so as to have an oval space of an inch and a half exposed. Iodoform was dusted over the entire surface with iodoform gauze placed in the opening and the whole covered with antiseptic sublimate gauze, over which a layer of absorbent cotton was secured by a bandage around the body.

The temperature and pulse showed slight increase during the following days, and the stitches were removed on December 10th, the adhesion being complete

On the 12th the patient was found with a pulse of 120 and temperature 104 degrees, having a profuse perspiration following rigors during the night. The wound was granulating, and in all respects was in good condition, but the tongue was much coated, and a discharge from the anus indicated disintegration of the rectal tumor. This aggravation of general condition was therefore attributed to septic contamination from the breaking down of the induration, having no connection whatever with the previous operation. The patient was put upon sulph. quin. grs. v, bic. soda grs. vi, calomel gr. i, in capsules every two hours until five doses were taken, which induced fecal evacuation.

In the meantime the rectum was washed out with a solution of boracic acid, and the mixture of iodoform with balsam of Peru left in the rectum. All local applications had been suspended after the operation, as there was no prospect of accomplishing any change in the tumor, but the above course was pursued daily from this time.

On the afternoon of the same day the temperature had fallen to 100 degrees and the pulse to 110 beats, and on the day following they became normal.

A combination of Huxam's tinct. of cinchona and serpentaria, tinct. nux vomica and chlorate of potash was given every two or three hours, and on the 14th his condition being favorable, preparations were made for completing the colotomy.

With the assistance of Drs. Divine, Elkin, Nicolson and Murray, Dr. Gaston completed the operation in the following manner:

Hypodermic of morphia and atropia was given; cocaine, 4 pc. solution, was applied to graulating wound; a tenaculum was secured in the coats of the intestine near each angle of the incision to lift it up and put it on the stretch, a small puncture being made with a knife at one end of the proposed line of opening, and a probe-pointed bistoury was pressed through this for the division of the walls of the colon to the extent of an inch and a quarter. Retractors were used on each side to draw the cut edges back, and a plug, made by tying oil silk around cotton, so as to bulge in the form of a ball, was pressed into the bowel and retained by a compress of gauze laid over the expanded corners of the oil silk. A fold of cotton was firmly secured over all by a broad roller bandage around the body. There was but little sensation of pain by the patient, and his general condition was good subsequently, under the continued use of half grain of gum opium at night. He has had the dressings renewed daily with copious fecal evacuations from the opening, and but little discharge from the anus. The boracic solution and iodoform, with Peruvian balsam, continue to be applied twice a day in the rectum for the correction of the disintegrating process in the tumor.

On the 9th the Huxam's tincture mixture was discontinued, and a combination of mur. tinct. iron and tinct. nux vomica ordered four times a day to counteract a tendency to night sweats. In all other respects the case is progressing satisfactorily, excepting that a swelling has occurred to the left of the anus, connected with the carcinomatous degeneration.

